# Electronic nose dataset for detection of wine spoilage thresholds

**DOI:** 10.1016/j.dib.2019.104202

**Published:** 2019-06-27

**Authors:** Juan C. Rodriguez Gamboa, Eva Susana Albarracin E., Adenilton J. da Silva, Tiago A. E. Ferreira

**Affiliations:** a Departamento de Estatística e Informática, Universidade Federal Rural de Pernambuco - UFRPE, Recife, PE, Brazil; b Centro de Informática, Universidade Federal de Pernambuco - UFPE, Recife, PE, Brazil

**Keywords:** Electronic nose, Chemical sensing, Machine learning, Beverage quality control, Wine spoilage

## Abstract

In this data article, we provide a time series dataset obtained for an application of wine quality detection focused on spoilage thresholds. The database contains 235 recorded measurements of wines divided into three groups and labeled as high quality (HQ), average quality (AQ) and low quality (LQ), in addition to 65 ethanol measurements. This dataset was collected using an electronic nose system (E-Nose) based on Metal Oxide Semiconductor (MOS) gas sensors, self-developed at the Universidade Federal Rural de Pernambuco (Brazil). The dataset is related to the research article entitled “Wine quality rapid detection using a compact electronic nose system: application focused on spoilage thresholds by acetic acid” by Rodriguez Gamboa et al., 2019. The dataset can be accessed publicly at the repository: https://data.mendeley.com/datasets/vpc887d53s/

**Table:** Specifications table

Subject	Food Science; Computer Science Applications; Signal Processing
Specific subject area	Wine quality assessment using electronic nose technology
Type of data	Text files
How data were acquired	By using an electronic nose system (E-Nose) based on six Metal Oxide Semiconductor (MOS) gas sensors (MQ-3, MQ-4, MQ-6; two of each type).
Data format	Raw data, time series data
Parameters for data collection	In each experiment was used a 1 ml sample to amass the volatiles during 30 seconds inside the concentration chamber. The recorded data for each measurement corresponds to 180 seconds with 18.5 Hz sample rate. Then, the sensors were exposed to clean air for 600 seconds after the sample presentation.
Description of data collection	We collected wine samples categorized into three spoilage thresholds: low-quality (LQ), average-quality (AQ), and high-quality (HQ). In addition, we collected ethanol measurements in concentrations of 1%, 2.5%, 5%, 10%, 15%, and 20% (v/v).
Data source location	Institution: Universidade Federal Rural de Pernambuco City/Town/Region: Recife, PE Country: Brazil Latitude and longitude (and GPS coordinates) for collected samples/data: Latitude: 8° 1′ 2.68″ Longitude 34° 56′ 52.211'' (Latitude: −8.017852 | Longitude: −34.94785)
Data accessibility	Repository name: Mendeley Data Data identification number: https://doi.org/10.17632/vpc887d53s.3 Direct URL to data: https://data.mendeley.com/datasets/vpc887d53s/
Related research article	J.C. Rodriguez Gamboa, E.S. Albarracin E., A.J. da Silva, L. L. de Andrade Lima, T.A. E. Ferreira, Wine quality rapid detection using a compact electronic nose system: application focused on spoilage thresholds by acetic acid, LWT - Food Science and Technology. 108 (2019) 377–384. https://doi.org/10.1016/j.lwt.2019.03.074.

**Table:** 

**Value of the data** • The dataset is available as a benchmark of E-Nose applications, focused on wine spoilage thresholds studies. • This dataset is useful for testing classifiers and pattern recognition methods with comparison purposes in studies related to E-Nose applications. • To the best of our knowledge, this dataset is the first one publicly available regarding commercial wines measurements acquired with E-Nose. • These data are suitable to support E-Nose applications, helping in the decision-making of winemakers and consumers in routine tasks of wine quality control [2].

## 1 Data

The recorded time series was acquired at the sampling frequency of 18.5 Hz during 180 seconds, resulting in 3330 data points per sensor. Each file in the dataset has eight columns: relative humidity (%), temperature (°C), and the resistance readings in kΩ of the six gas sensors: MQ-3, MQ-4, MQ-6, MQ-3, MQ-4, MQ-6.

We organized the database in three folders for the wines: AQ_Wines, HQ_Wines, LQ_Wines, and one folder for the ethanol. Each folder contains text files that correspond to different measurements.

In the wines folders, each filename identifies a wine measurement as follows: the first 2 characters of the filename are an identifier of the spoilage wine threshold (AQ: average-quality, HQ: high-quality, LQ: low-quality); characters 4–9 indicate the wine brand; characters 11–13 indicate the bottle, and the last 3 characters indicate the repetition (another sample of the same bottle). For example, file LQ_Wine01-B01_R01 contains the time series recorded when low-quality wine of the brand 01, bottle 01, sample 01 was measured.

In the Ethanol folder, each filename identifies an ethanol measurement as follows: the first 2 characters of the filename are an identifier of Ethanol (Ea); characters 4–5 indicate the concentration in v/v (C1: 1%, C2: 2.5%, C3: 5%, C4: 10%, C5: 15%, C6: 20%); and the last 3 characters indicate the repetition. For example, file Ea-C1_R01 contains time series acquired when Ethanol at 1% v/v of concentration, sample 01 was measured.

**Fig. 1:**
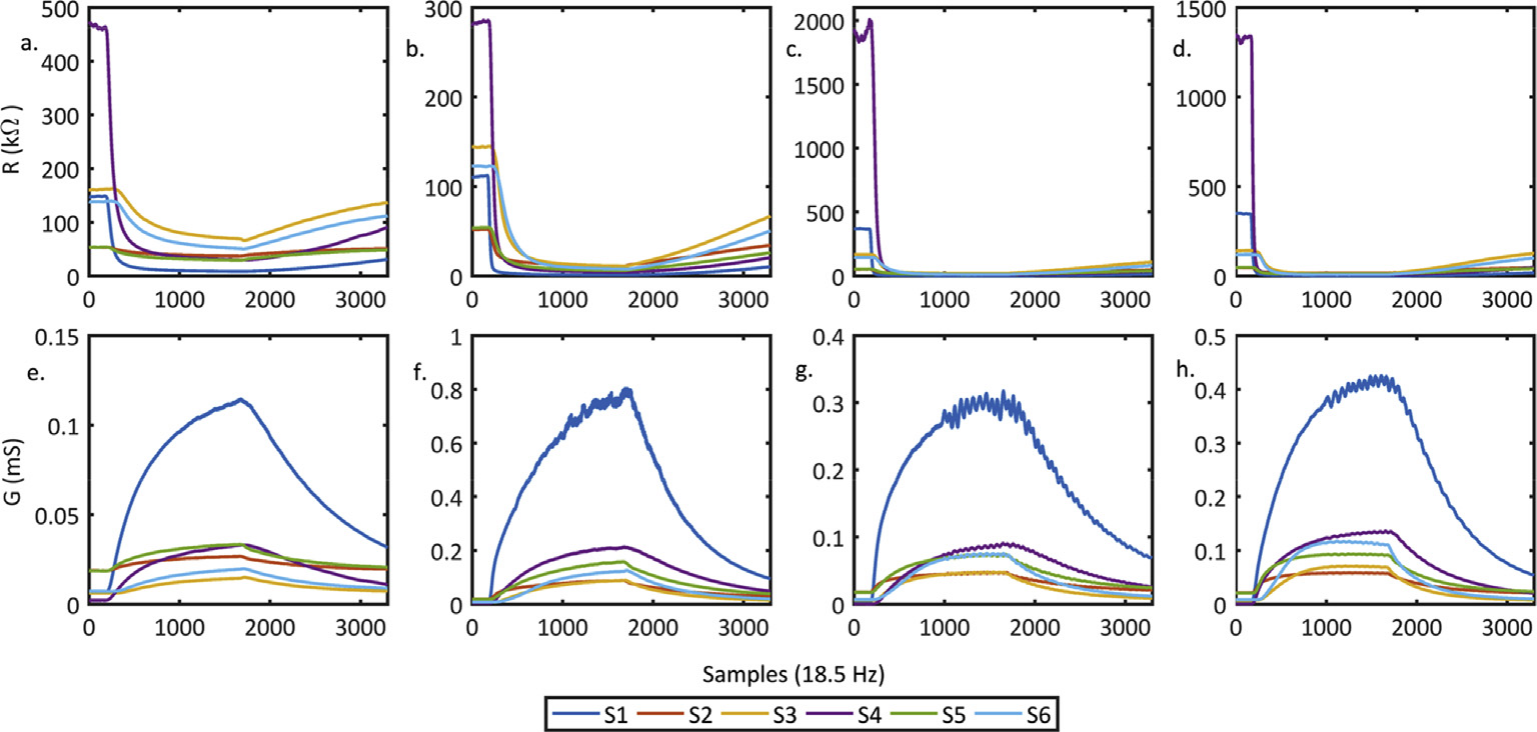
Measurements acquired with our E-Nose, where S1, S2,…, S6 represent the gas sensors outputs; a. and e. correspond to the dataset file EaC1R10 (ethanol measurement); b. and f. correspond to LQWine02B01R09 dataset file; c. and g. correspond to AQWine01B01R07 dataset file; d. and h correspond to HQWine05B01R01 dataset file.

In Fig. 1, we depicted the time series for several measurements collected in this work. The measurements displayed at the top of the figure are in resistance units (Ω), and at the bottom side are the same measurements in conductance units (S).

## 2 Experimental design, materials, and methods

### 2.1 Experimental setup

**Fig. 2:**
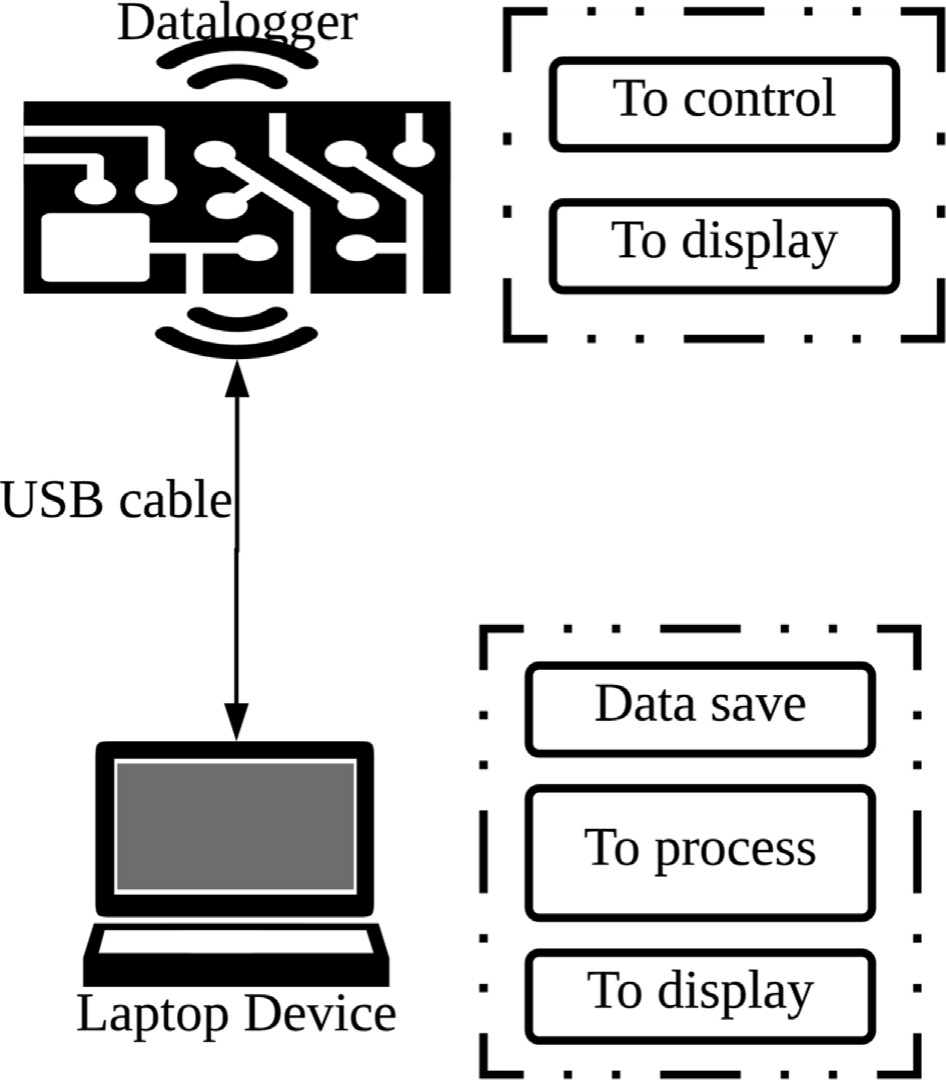
Operating general diagram of O-NOSE system.

The dataset was collected with an E-Nose self-developed, that was named O-NOSE. We designed the datalogger for operating linked to a computer that has the proper software for data recording and processing, as shown in Fig. 2.

**Fig. 3:**
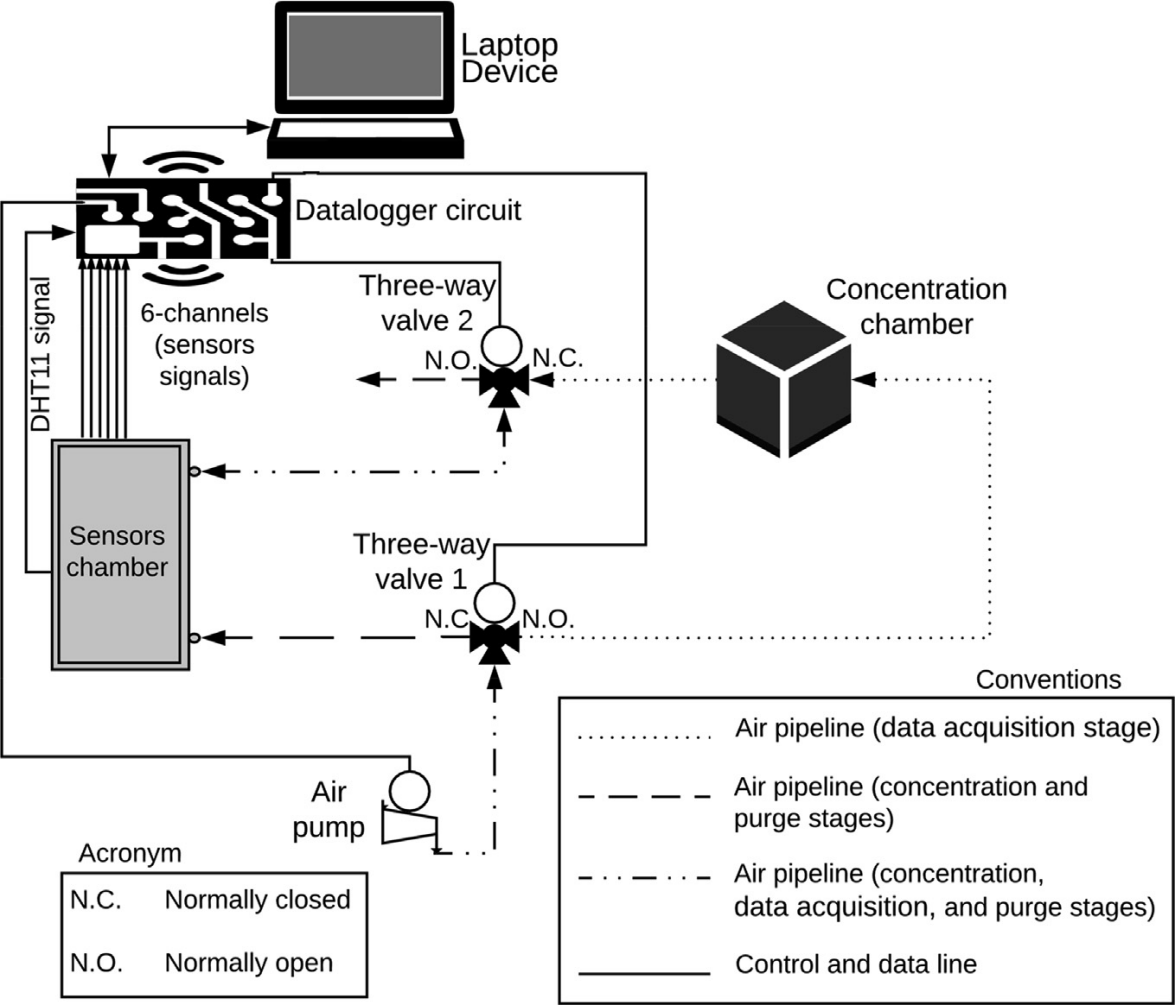
Schematic diagram of O-NOSE displaying the operation stages.

The operating mode of O-NOSE is depicted with more details in Fig. 3. The device contains two mini three-way solenoid valves ZHV 0519, and two mini air pumps PM201U (these actuators work with +5 VDC in the same way of all elements in the system) controlled by an embedded device: microcontroller Arduino Nano. The microcontroller takes charge of the data acquisition from the gas sensors and the temperature and humidity sensor DHT11 located into the sensors chamber. As well, of the timing control of the solenoid valves and the air pump, and the communication with the computer.

It used a 100 ml concentration chamber, where is placed the specimen to be analyzed. The sensor array of six MOS gas sensors manufactured by Hanwei Sensors (MQ-3, MQ-4, and MQ-6; two of each) is located into a 200 ml chamber connected to pneumatic hoses that carry the volatiles. The gas is sensed by its effect on the sensitive layer of tin dioxide (SnO_2_), resulting from changes in conductivity brought about by chemical reactions on the surface of the tin dioxide particles [3], [4].

The stages of the measurement process are concentration, data acquisition, and purge. The first stage aims to accumulate the analyte volatiles inside the concentration chamber for 30 seconds, to achieve this, the microcontroller activates the valve 1 and the air pump; simultaneously, deactivates the valve 2 for isolating the concentration chamber interior of the external environment. In Fig. 3, the dashed line indicates the airflow at this stage.

The data acquisition stage that lasts 3 min aims to collect the signals from the gas sensors, to achieve this, the microcontroller deactivates the valve 1 and activates the valve 2 and the air pump to direct the airflow from the concentration chamber dragging the volatiles towards the sensors chamber. In Fig. 3, the dotted line indicates the airflow at this stage.

The goal of the purge stage is to clean and remove volatile residues from the previous measurement during 10 minutes. Hence, the microcontroller activates the valve 1 and the air pump; simultaneously, deactivates the valve 2, the same way that for concentration stage. In Fig. 3, the dashed line indicates the airflow at this stage.

### 2.2 Measurement protocol

O-NOSE performs the measurement process in three stages: concentration, data acquisition (the recorded data corresponds to 180 seconds with 18.5 Hz sample rate) and purge [1]. Each measurement corresponds to the time-dependent output voltages of each gas sensor converted to resistance values according to the voltage-divider scheme [5] and the corresponding load resistor ($R_{L}$). The sensor resistance ($R_{S}$) value changes when the gas sensor is exposed to a certain specimen and was calculated as follows:

(1) $$R_{s}=\frac{V_{C}-V_{R_{L}}}{V_{R_{L}}}\times R_{L}$$

(2) $$V_{R_{L}}=\frac{ADC\times V_{C}}{1023}$$

where $V_{C}$ , $V_{R_{L}}$, $R_{L}$, ADC are the standard voltage of microcontroller (5V), the output voltage, sensor load resistor, and the Analog to Digital Converter (ADC) reading, respectively [5].

### 2.3 Samples

We used 22 bottles of commercial wines of different varieties and vintages, elaborated in four wineries of the São Francisco valley (Pernambuco-Brazil). To obtain spoiled samples, 13 of the 22 bottles were randomly selected and left opened for six months before starting the measurements (low-quality LQ wines). Besides, four bottles were opened two weeks before beginning the data collection (average-quality AQ wines), and the remaining five bottles were opened at the starting time of each measurement (high-quality HQ wines) [1].

In addition to wines, we measured isolated ethanol in concentrations (v/v): 2, 5, 10, 20, 30, and 40 ml of ethanol diluted in distilled water to make solutions of 200 ml. These concentrations allow guaranteeing a range that covers the different possible values in wines with and without spoilage. To ensure the repeatability of the experiments using O-NOSE, we collected between 10 and 11 samples of 1mL of each wine bottles; and between 10 and 12 of the ethanol samples at their different concentrations. In this way, the database contains 235 measurements of wines divided into three groups: high quality (HQ), average quality (AQ) and low quality (LQ), with 51, 43, and 141 measurements, respectively, and 65 ethanol measurements [1].
